# Risk factors associated with *Candida auris* Candidemia: A single-centre retrospective case–control study

**DOI:** 10.1017/S0950268825100721

**Published:** 2025-11-03

**Authors:** Ayse Kaya, Fatma Eser, Imran Hasanoglu, Bircan Kayaaslan, Aziz Bozkurt, Ayse Yasemin Tezer Tekce, Bedia Dinc, Fisun Kirca, Hatice Rahmet Guner

**Affiliations:** 1 Infectious Diseases and Clinical Microbiology, Ankara City Hospital, Ankara Yildirim Beyazit University, Ankara, Türkiye; 2 Infectious Diseases and Clinical Microbiology, Ankara City Hospital, Ankara, Türkiye; 3 Infectious Diseases and Clinical Microbiology, Ministry of Health, Ankara Bilkent City Hospital, Ankara, Türkiye; 4 Medical Microbiology, Ministry of Health, Ankara City Hospital, Ankara, Türkiye; 5 Medical Microbiology, Ankara Bilkent City Hospital, Ankara, Türkiye

**Keywords:** antifungal resistance, *Candida auris*, *Candidozyma auris*, critical care, risk factors

## Abstract

*Candida auris* has emerged as a major nosocomial pathogen due to multidrug resistance (MDR), outbreak potential, and high mortality in critically ill patients. Identifying risk factors for *C. auris* candidemia is essential for prevention and infection control. In this single-centre, retrospective case–control study, we analysed adults with *C. auris* candidemia (n = 52) and matched controls (n = 104) hospitalized between February 2019 and October 2024. Matching was based on hospital unit and blood culture timing. Clinical and epidemiological variables were compared, and multivariate logistic regression identified independent risk factors. Antifungal susceptibility and 14- and 28-day all-cause mortality were evaluated as secondary outcomes. Independent risk factors included recent hospitalization (odds ratio (OR): 7.93), prolonged hospital stay (OR: 1.01), prior broad-spectrum antibiotic use (OR: 46.20), central venous catheter (CVC) (OR: 3.88), sepsis (OR: 9.43), and high Candida Colonization Index (OR: 14.10). All-cause mortality at 14 and 28 days was 30.8% and 46.2%, respectively. Fluconazole resistance was 96%, while 8.7% of isolates were pandrug resistant. *C. auris* candidemia represents a serious clinical challenge with substantial mortality and modifiable risk factors. Strengthening antimicrobial stewardship, colonization surveillance, and early recognition in high-risk patients may reduce its impact.

## Introduction

First identified in 2009, *Candida/Candidozyma auris* has rapidly spread worldwide, causing outbreaks in numerous healthcare settings [1, 2]. Despite the implementation of comprehensive infection control measures, its eradication remains extremely challenging. Due to its phenotypic similarity to other Candida species and phylogenetic proximity, accurate microbiological identification is often difficult [3–5].

*C. auris* is notable for its multidrug resistance (MDR) to commonly used antifungals, its ability to persistently contaminate hospital environments, its resistance to broad-spectrum disinfectants, and its capacity to cause outbreaks in intensive care units (ICUs). It has been associated with bloodstream infections in critically ill patients and is linked to high crude mortality rates [1, 6–16]. Owing to these characteristics, it was classified as an emerging multidrug-resistant fungal pathogen by the Centers for Disease Control and Prevention (CDC) in 2016 and by the World Health Organization (WHO) in 2022 [17, 18]. To date, *C. auris* has been isolated in more than 47 countries across six continents. Major risk factors for colonization and infection include prolonged exposure to healthcare settings, the presence of multiple comorbidities, and invasive medical procedures [19–22].

To the best of our knowledge, case–control studies evaluating risk factors for *C. auris* candidemia are limited in the literature, particularly from Turkey. This study aims to address that gap by providing comparative data between infected and non-infected hospitalized patients. Furthermore, the findings of our study may facilitate early diagnosis and timely initiation of appropriate antifungal therapy. They may also contribute to strengthening national infection control strategies and preventing invasive infections caused by *C. auris.*

## Methods

### Study aim, design, and setting

This study was designed as a single-centre, retrospective, case–control study. The study period was from 25 February 2019 to 21 October 2024. Adult patients with at least one blood culture positive for *C. auris* were included in the case group. Only the first episode of *C. auris* candidemia was considered for each case. The cases and controls were matched in a 1:2 ratio. The matching criteria included random selection, admission to the same unit as the case, and having a blood culture obtained within a 15-day window (within 7 days before or 7 days after the date of the case’s positive blood culture). Control patients were included only if their blood cultures showed no microbial growth. The primary outcome was to determine the risk factors associated with the development of candidemia. In the case group, antifungal susceptibility results, as well as 14-day and 28-day all-cause mortality, were defined as secondary outcomes.

The study was approved by the Ankara City Hospital Ethical Committee (TABED 1-24-703).

### Description of materials

Data were retrospectively collected from electronic medical records. Variables recorded included age, sex, hospital and ICU admission dates, presence of comorbidities, and the hospital unit where the patient was staying at the time the blood culture was drawn. The Charlson Comorbidity Index and Sequential Organ Failure Assessment (SOFA) score were calculated for all patients, and the Acute Physiology and Chronic Health Evaluation II (APACHE II) score was calculated for patients admitted to the ICU. In addition, to evaluate potential risk factors, recent hospitalization, recent abdominal surgery, prior use of broad-spectrum antibiotics or antifungal agents, length of hospital and ICU stay, mechanical ventilation (MV), presence of a central venous catheter (CVC), total parenteral nutrition (TPN) therapy, and neutropenia were recorded. The presence of sepsis at the time of study inclusion was assessed.

Candida colonization, multisite Candida colonization, *C. auris* colonization, multisite *C. auris* colonization, and the presence of any *C. auris*–colonized patient in the same hospital unit were determined. The Candida Colonization Index was calculated as the ratio of the number of colonized sites to the total number of sites cultured; a value ≥0.5 was considered significant for predicting invasive infection [23]. The Candida score was determined according to León et al., incorporating factors such as multifocal colonization, surgery, parenteral nutrition, and severe sepsis; a score ≥3 was considered predictive of candidemia [24]. In the case group, antifungal susceptibility results were recorded, while 14-day and 28-day all-cause mortality was calculated from the date of the first positive blood culture.

### Definitions

Comorbidities were defined as follows: chronic cardiac diseases (including hypertension, coronary artery disease, heart failure, arrhythmias, and history of valve surgery), diabetes mellitus, chronic neurological diseases (such as Alzheimer’s disease, Parkinson’s disease, or history of cerebrovascular events), immunodeficiency (including active solid organ or hematologic malignancy, or human immunodeficiency virus (HIV) infection), chronic kidney disease (defined as glomerular filtration rate (GFR) <60 mL/min/1.73 m^2^), dialysis treatment, and other conditions such as chronic obstructive pulmonary disease, liver cirrhosis, chronic hepatitis B, benign prostatic hyperplasia, and hyperthyroidism.

Recent hospitalization was defined as a hospital stay of at least 5 days within the previous 3 months, and recent abdominal surgery was defined as a history of abdominal surgery within the past 1 month. Use of broad-spectrum antibiotics or antifungal agents was considered a risk factor if administered within the 7 days prior to study inclusion. Length of hospital and ICU stay was defined as the time from hospital or ICU admission to the onset of *C. auris* candidemia in the case group and to the date of inclusion in the control group for controls. The presence of CVC, TPN, MV, and neutropenia was considered a potential risk factor and was defined as having occurred for at least 48 h within the 7 days prior to study inclusion.

Candida colonization was defined as the isolation of *Candida* species (excluding *C. auris*) from at least one non-sterile site (e.g. urine, skin, and/or respiratory tract specimens) in the absence of clinical signs or symptoms of infection. Multisite Candida colonization was defined as the isolation of *Candida* species (excluding *C. auris*) from more than one non-sterile site.

*C. auris* colonization was defined as the isolation of *C. auris* from at least one non-sterile site (urine, skin, and/or respiratory tract specimens) in the absence of clinical signs or symptoms of infection. Multisite *C. auris* colonization was defined as isolation from more than one non-sterile site. A low Candida Colonization Index was defined as a Candida Colonization Index <0.5, and a high Candida Colonization Index was defined as a Candida Colonization Index ≥0.5.

Resistance to two of the following antifungal agents – fluconazole, amphotericin B, and echinocandins (caspofungin or micafungin) – was defined as MDR, while resistance to all three was defined as pandrug resistance (PDR) [25].

### Microbiological analysis

Laboratory data including antifungal susceptibility results were extracted from the laboratory information management system. Identification of isolates was done using matrix-assisted laser desorption/ionization time-of-flight (MALDI-TOF) mass spectrometry (Bruker, Billerica, USA). Antifungal susceptibility testing was performed by two different approaches depending on the study period. Before September 2023, isolates were sent to the National Reference Laboratory for testing, where antifungal susceptibility was determined using the broth microdilution method, and available results were retrieved from the national public health database. After that date, testing was performed in our hospital laboratory using the Sensititre YeastOne System (Thermo Fisher Scientific, USA), which is based on the broth microdilution method. Antifungal susceptibility testing could not be performed for all isolates because some stored strains were not available at the time of testing. Minimum inhibitory concentrations (MICs) of antifungals were interpreted using the CDC tentative interpretive breakpoints [26, 27].

### Statistical analysis

Statistical analyses were performed using MedCalc® Statistical Software version 22.009 (MedCalc Software Ltd., Ostend, Belgium; https://www.medcalc.org; 2023). Continuous variables were summarized as median (min–max) values due to non-normal distribution. Categorical variables were summarized as frequency (n) and percentage (%).

Appropriate hypothesis tests were applied to assess relationships between groups. The chi-square test was used for the analysis of categorical variables, and Fisher’s exact test or likelihood ratio test was employed when appropriate. The Shapiro–Wilk test was used to assess the normality of distribution. For comparisons between two independent variables not normally distributed, the Mann–Whitney U test was applied.

The effect of potential risk factors on *C. auris* infection was evaluated using univariate and multivariate logistic regression analyses. A p-value of <0.05 was considered statistically significant in all analyses. A post hoc power analysis was conducted based on the significant variables identified in the multivariate model.

## Results

A total of 52 patients were included in the case group and 104 patients in the control group. The demographic and clinical characteristics and risk factors of the patients are presented in Table 1. The mean age of the cases was 65.2 ± 19.9 years, and 57.7% (n = 30) were over 65 years of age. There was no significant difference between the case and control groups in terms of age (65.2 ± 19.9 vs. 66.5 ± 17.0; p = 0.970) or sex (male: 65.4% vs. 53.8%; p = 0.744). At least one comorbid condition was present in 88.2% (n = 45) of the cases and 92.2% (n = 95) of the controls (p = 0.552). The most common comorbidities were chronic heart disease (63.5% vs. 56.7%; p = 0.744), diabetes mellitus (36.5% vs. 30.8%; p = 0.333), and chronic neurological disease (32.7% vs. 23.1%; p = 0.331). The mean Charlson Comorbidity Index (4.3 ± 2.8 vs. 4.3 ± 2.1; p = 0.838) and SOFA scores (8.4 ± 4.7 vs. 6.9 ± 5.1; p = 0.103) were similar between the groups. However, the mean APACHE II score was significantly higher in the control group (16.8 ± 9.3 vs. 20.3 ± 10; p = 0.036). The distribution of hospital units where patients were admitted was similar in both groups, with the majority being in ICUs (84.6% vs. 88.5%; p = 0.108).

**Table 1.** Demographic and clinical characteristics and risk factors of patients in case and control groups

	Case group n = 52 (%)	Control group n = 104 (%)	*P*
Age, years, median (min–max)	69 (21–94)	69 (19–96)	0.970^a^
>65 years	30 (57.7)	60 (57.7)	1.00^b^
Sex, male	34 (65.4)	56 (53.8)	0.744
Presence of comorbidity	45 (88.2)	95 (92.2)	0.552^c^
Chronic cardiac diseases	33 (63.5)	59 (56.7)	0.744
Diabetes mellitus	19 (36.5)	32 (30.8)	0.333
Chronic neurological diseases	17 (32.7)	24 (23.1)	0.331
Immunodeficiency	12 (23.07)	22 (21.15)	0.251
Chronic kidney disease	11 (21.15)	10 (9.61)	0.589
Dialysis	8 (15.38)	12 (11.53)	0.867
Others	9 (17.29)	27 (25.94)	0.331
Charlson Comorbidity Index (n = 50, 102) median (min–max)	4 (0–13)	4 (0–9)	0.838^a^
APACHE II score (n = 44, 92) median (min–max)	15 (3–51)	19 (3–48)	**0.036** ^a^
SOFA score (n = 52, 76) median (min–max)	8 (0–21)	7 (0–19)	0.103^a^
Hospital unit			0.108
ICU	44 (84.6)	92 (88.5)	
Floor	6 (11.5)	12 (11.5)	
Emergencies	2 (3.80)	0	
Recent hospitalization	18 (34.6)	13 (12.5)	**0.002** ^b^
Recent abdominal surgery	2 (3.8)	4 (3.8)	1.00^c^
Length of hospital stay (days) (n = 152, 104) median (min–max)	45.5 (0–232)	14 (0–391)	**<0.001** ^a^
Length of ICU stay (days) (n = 44, 92) median (min–max)	38 (0–173)	10 (1–387)	**<0.001** ^a^
Prior use of broad-spectrum antibiotics	51 (98.1)	73 (70.2)	**<0.001** ^b^
Prior use of antifungal agents	14 (26.9)	15 (14)	0.094^b^
MV	33 (63.5)	60 (57.7)	0.604^b^
Neutropenia	1 (1.9)	3 (2.9)	1.00^c^
TPN	15 (29.4)	20 (19.2)	0.222^b^
CVC	39 (75)	55 (52.9)	**0.013** ^b^
Sepsis	12 (23.1)	9 (8.7)	**0.025** ^b^
Candida colonization	20 (43.5)	37 (37)	0.574^b^
Multisite candida colonization	8 (38.1)	6 (16.2)	0.121^b^
Candida Colonization Index (n = 46, 100) median (min–max)	0 (0–1)	0 (0–0.7)	0.120^a^
Low Candida Colonization Index	12 (26.1)	32 (32)	0.597^b^
High Candida Colonization Index	9 (18.6)	5 (5)	**0.012** ^c^
Candida score (n = 46, 100) median (min–max)	1 (0–5)	1 (0–5)	0.505^a^
*C. auris* colonization	35 (68.6)	20 (19.8)	**<0.001** ^b^
Multisite *C. auris* colonization	14 (27.5)	2 (2.5)	**<0.001** ^b^
Presence of *C. auris*–colonized patient in same unit	24 (46.2)	57 (54.8)	0.395^a^
14-day mortality	16/52 (30.8)	21/102 (20.6)	
28-day mortality	24/52 (46.2)	35/102 (34.3)	

Abbreviations: APACHE II: acute physiology and chronic health evaluation II; CVC: central venous catheter; ICU: intensive care unit; MV: mechanical ventilation; SD: standard deviation; SOFA: sequential organ failure assessment; TPN: total parenteral nutrition.

a Mann–Whitney U test.

b Yates’ continuity correction.

c Fisher’s exact test.

Risk factors were compared between the case and control groups (Table 1). A recent abdominal surgery was present in 3.8% of both groups (p = 1). Recent hospitalization (34.6% vs. 12.5%; p = 0.002), length of hospital stay (57 ± 51.8 vs. 34.2 ± 2.6 days; p < 0.001), and length of ICU stay (47.2 ± 38 vs. 27.2 ± 64.1 days; p < 0.001) were significantly higher in the case group. *C. auris* candidemia was identified at a median of 45.5 days after hospital admission and 38 days after ICU admission.

Broad-spectrum antibiotic use was significantly more common in the case group (98.1% vs. 70.2%; p < 0.001), whereas prior antifungal use was not statistically different (26.9% vs. 14%; p = 0.094). The presence of MV, neutropenia, and TPN therapy did not differ significantly between the groups (MV: 63.5% vs. 57.7%; p = 0.604, neutropenia: 1.9% vs. 2.9%; p = 1, TPN: 29.4% vs. 19.2%; p = 0.222). However, the presence of a CVC (75% vs. 52.9%; p = 0.013) and sepsis (23.1% vs. 8.7%; p = 0.025) was significantly more frequent in the case group.

There were no significant differences between the groups in terms of *Candida* colonization (43.5% vs. 37%; p = 0.574), multisite *Candida* colonization (38.1% vs. 16.2%; p = 0.121), mean Candida Colonization Index (0.2 ± 0.3 vs. 0.1 ± 0.2; p = 0.120), mean *Candida* score (1.2 ± 1.1 vs. 1.0 ± 1.1; p = 0.505), or the presence of a *C. auris*–colonized or infected patient in the same hospital unit (46.2% vs. 54.8%; p = 0.395). Compared to the control group, the case group had significantly higher rates of patients with high Candida Colonization Index (18.6% vs. 5%; p = 0.012), *C. auris* colonization (68.6% vs. 19.8%; p < 0.001), and multisite *C. auris* colonization (27.5% vs. 2.5%; p < 0.001).

Risk factors associated with the development of *C. auris* candidemia were analysed using logistic regression (Table 2). According to the univariate analysis, the following were identified as significant risk factors: total length of hospital stay (odds ratio (OR): 1.01, 95% confidence interval (CI): 1.00–1.01, *p* = 0.041), recent hospitalization (OR: 3.71, 95% CI: 1.64–8.37, *p* = 0.002), prior use of broad-spectrum antibiotics (OR: 21.66, 95% CI: 2.86–163.78, *p* = 0.003), presence of a CVC (OR: 2.67, 95% CI: 1.28–5.58, *p* = 0.009), presence of sepsis (OR: 3.17, 95% CI: 1.24–8.11, *p* = 0.016), *C. auris* colonization (OR: 8.86, 95% CI: 4.11–19.09, *p* < 0.001), multisite *C. auris* colonization (OR: 14.95, 95% CI: 3.23–69.17, *p* < 0.001), mean Candida Colonization Index (OR: 5.19, 95% CI: 1.24–21.83, *p* = 0.025), and high Candida Colonization Index (OR: 4.62, 95% CI: 1.45–14.70, *p* = 0.010). In the multivariate analysis (Table 2), the strongest independent predictors of *C. auris* candidemia were prolonged hospital stay (OR: 1.01, 95% CI: 1.00–1.03, *p* = 0.007), recent hospitalization (OR: 7.93, 95% CI: 2.00–31.40, *p* = 0.003), prior use of broad-spectrum antibiotics (OR: 46.20, 95% CI: 2.90–734.70, *p* = 0.007), presence of a CVC (OR: 3.88, 95% CI: 1.02–14.81, *p* = 0.047), presence of sepsis (OR: 9.43, 95% CI: 1.60–55.45, *p* = 0.013), and high Candida Colonization Index (OR: 14.10, 95% CI: 1.45–137.06, *p* = 0.023). Although included in the model, prior antifungal use, TPN, and *C. auris* colonization were not significantly associated with candidemia development. Post hoc power analysis showed power values of 88.5% for recent hospitalization, 99.9% for prior use of broad-spectrum antibiotics, 77.3% for presence of a CVC, 68% for presence of sepsis, and 78.4% for high Candida Colonization Index.

**Table 2.** Univariable and multivariable analyses of factors associated with the development of *C. auris* candidemia

	Univariable analysis		Multivariable analysis^a^	
	Odds ratio (95% CI)	*P*	Odds ratio (95% CI)	*P*
Age, years	0.99 (0.98–1.01)	0.653		
>65 years	1.00 (0.51–1.96)	1.00		
Presence of comorbidity	0.63 (0.21–1.93)	0.420		
Charlson comorbidity index	1.01 (0.87–1.17)	0.910		
APACHE II score	0.96 (0.93–1.00)	0.055		
SOFA score	1.07 (0.99–1.15)	0.088	1.05 (0.92–1.20)	0.432
Hospital unit; ICU	1.05 (0.37–2.97)	0.933		
Recent hospitalization	**3.71 (1.64–8.37)**	**0.002**	**7.93 (2.00–31.40)**	**0.003**
Length of hospital stay	**1.01 (1.00–1.01)**	**0.041**	**1.01 (1.00–1.03)**	**0.007**
Length of ICU stay	1.01 (0.99–1.01)	0.085		
Prior use of broad-spectrum antibiotics	**21.66 (2.86–163.78)**	**0.003**	**46.20 (2.90–734.70)**	**0.007**
Prior use of antifungal agents	2.19 (0.96–4.97)	0.062	0.21 (0.04–1.02)	0.052
MV	1.27 (0.64–2.53)	0.489		
Neutropenia	0.66 (0.07–6.51)	0.722		
TPN	1.75 (0.81–3.80)	0.157	2.71 (0.78–9.46)	0.118
CVC	**2.67 (1.28–5.58)**	**0.009**	**3.88 (1.02–14.81)**	**0.047**
Sepsis	**3.17 (1.24–8.11)**	**0.016**	**9.43 (1.60–55.45)**	**0.013**
Candida colonization	1.31 (0.64–2.67)	0.457		
Multisite candida colonization	3.18 (0.92–10.99)	0.068		
Candida Colonization Index	**5.19 (1.24–21.83)**	**0.025**		
Low Candida Colonization Index	0.75 (0.34–1.64)	0.470		
High Candida Colonization Index	**4.62 (1.45–14.70)**	**0.010**	**14.10 (1.45–137.06)**	**0.023**
Candida score	1.11 (0.82–1.50)	0.502		
*C. auris* colonization	**8.86 (4.11–19.09)**	**<0.001**	-	0.997
Multisite *C. auris* colonization	**14.95 (3.23–69.17)**	**<0.001**		
Presence of *C. auris*–colonized patient in same unit	0.71 (0.36–1.38)	0.309		

Abbreviations: APACHE II, acute physiology and chronic health evaluation II; CVC, central venous catheter; ICU, intensive care unit; MV, mechanical ventilation; SOFA, sequential organ failure assessment; TPN, total parenteral nutrition.

a Only the variables that remained in the final multivariable analysis are reported with their corresponding odds ratios and 95% confidence intervals.

Antifungal susceptibility and MIC results of the isolates are presented in Table 3. Antifungal susceptibility testing was not performed for all strains. Among the tested isolates, resistance rates were 96% for fluconazole, 18.2% for amphotericin B, 8% for caspofungin, and 0% for micafungin. Susceptibility results for fluconazole, amphotericin B, and echinocandins were available for 23 isolates. Based on these, the rate of MDR was 13% (3), and the rate of PDR was 8.7% (2). All-cause mortality at 14 and 28 days was 30.8% (16/52) and 46.2% (24/52), respectively. Among 25 patients with available antifungal susceptibility results, antifungal therapy was initiated on the day of *C. auris* candidemia diagnosis in 6 patients (24%) and within 24 h after blood culture collection in 14 patients (56%). All 20 patients who received antifungal therapy were treated with agents to which their isolates were susceptible according to antifungal susceptibility testing. In these 20 patients, the 14-day and 28-day all-cause mortality rates were 30% (6/20) and 55% (11/20), respectively.

**Table 3.** Antifungal susceptibility, resistance rates, and MIC distributions of the isolates

	Resistance (%) (MIC range)	Susceptibility (%) (MIC range)
Fluconazole, n = 25	24 (96) (128−>256)	1 (4) (4)
Amphotericin B, n = 22	4 (18.2) (2−>8)	18 (81.8) (0.5–1)
Caspofungin, n = 25	2 (8) (4−>8)	23 (92) (0.12–0.50)
Micafungin, n = 23	0	23 (100) (0.06–0.25)

Abbreviation: MIC, Minimum inhibitory concentration.

## Discussion

The increasing global incidence of *C. auris*, along with its association with healthcare-associated outbreaks and rising mortality rates, has made it a significant public health concern. The findings of this study suggest that identifying risk factors associated with *C. auris* may contribute not only to the improvement of early diagnosis and treatment strategies but also to the development of preventive measures targeting modifiable risk factors.

As a result of our study, recent hospitalization history, prolonged hospital stay, use of broad-spectrum antibiotics, presence of a CVC, sepsis, and a high Candida Colonization Index were found to be significantly associated with *C. auris* candidemia. Similarly, the literature has reported various clinical conditions as potential risk factors for *C. auris* infection or colonization, including the presence of a CVC, tracheostomy, percutaneous endoscopic gastrostomy (PEG), MV, prior antibiotic use, ICU admission, diabetes mellitus, and history of abdominal surgery [28–35]. However, a significant proportion of these studies are case series or retrospective descriptive analyses, drawing conclusions primarily from characteristics frequently observed among affected patients. In contrast, our study utilized a case–control design, which allowed for the identification of independent risk factors by comparing patients with *C. auris* candidemia to matched controls without infection. Unlike descriptive or case series studies, this approach provides stronger epidemiological evidence by accounting for confounding variables and enabling statistical adjustment. Therefore, our findings offer more robust insight into potential causal relationships associated with *C. auris* candidemia. In a case–control study conducted in Colombia (focused on non–*C. auris* candidemia), ICU stay longer than 15 days, diabetes mellitus, and severe sepsis were reported as significant risk factors for *C. auris* candidemia. A key distinction of that study is that 41% of the patients were aged 0–18 years, and the comparison was made using a case–case design between *C. auris* and non–*C. auris* candidemia cases [36]. In our study, *C. auris* candidemia was observed in two patients upon admission to the emergency department. However, both had a recent history of hospitalization; therefore, these cases were not considered community-acquired infections. Interestingly, the mean APACHE II score was significantly higher in the control group. As this study aimed to identify risk factors for *C. auris* candidemia rather than assess clinical outcomes such as mortality, this unexpected finding may reflect unmeasured differences in baseline clinical severity among control patients. Importantly, it does not affect the interpretation of candidemia-associated risk factors.

*C. auris* spreads within healthcare settings either directly from colonized or infected patients to others or indirectly via the hands of healthcare workers and contaminated environmental surfaces [37–39]. It is considered a pathogen exclusively acquired in healthcare environments and has not been reported as a cause of community-acquired infections [40, 41]. Therefore, patients colonized with *C. auris* during hospitalization or at the time of admission are expected to be at increased risk for developing invasive infection. In our study, *C. auris* candidemia developed in 63.6% (35/55) of *C. auris*–colonized patients. To our knowledge, there are no published data from Turkey specifically evaluating the rate of *C. auris* candidemia among colonized patients; therefore, direct comparison with local studies is not possible. In univariate analysis, colonization with *C. auris* was associated with an 8.86-fold increased risk of candidemia (95% CI: 4.11–19.09); however, this association was not statistically significant in multivariate analysis. We initially considered that this could be explained by a potential overlap with the Candida Colonization Index, which reflects the extent of colonization. However, Candida colonization and *C. auris* colonization showed only a very small, non-significant association (φ = 0.10, p = 0.23; n = 145). To minimize conceptual overlap, Candida colonization was defined as the isolation of Candida species (excluding *C. auris*) from at least one non-sterile site (e.g. urine, skin, or respiratory specimens) in the absence of clinical signs or symptoms of infection. Therefore, both variables were not included simultaneously in the model, mainly to avoid conceptual overlap rather than true statistical collinearity. Since both variables are related to colonization burden, this conceptual overlap may still have influenced the independent predictive value of *C. auris* colonization in the regression model.

According to the literature, the cumulative incidence of candidemia among colonized patients has been reported as 25% within 60 days [1, 42, 43]. One study identified multisite *C. auris* colonization as the only independent predictor for progression to infection [43]. These findings suggest that not only the presence of colonization but also factors such as colonization burden, host immune status, and concurrent invasive procedures may play a role in the transition from colonization to infection. Nevertheless, it must be emphasized that colonized patients represent a major reservoir and focal point in healthcare-associated outbreaks [1, 44].

In our study, the 14-day and 28-day all-cause mortality rates were 30.8% and 46.2%, respectively. While mortality was not a primary outcome, the 28-day rate was notably high and reflects the severity of *C. auris* candidemia in critically ill patients. These findings support the need for early detection and appropriate antifungal management, particularly in high-risk populations. Crude mortality rates associated with *C. auris* have been reported in the literature to range from 23% to as high as 60% [19, 45–49]. These variations may be attributed to differences in hospital settings and patient characteristics. Our institution provides long-term healthcare and nursing services, which may influence outcomes. In our cohort, antifungal therapy was initiated on the day of diagnosis in 6 patients (24%) and at least 24 h after blood culture collection in 14 patients (56%). Interestingly, early initiation of antifungal therapy was not associated with lower mortality in this small subgroup, likely reflecting the greater severity of illness among patients who required immediate treatment. Therefore, while prompt recognition and management of *C. auris* candidemia remain essential, the present findings should be interpreted with caution and do not allow conclusions regarding the impact of treatment timing on survival.

In previous studies, factors associated with improved survival included age under 65 years, absence of comorbidities, hospital stay shorter than 3 months, absence of candidemia, appropriate antifungal therapy, absence of CVC use, no history of abdominal surgery, and no deep-seated candida complications [49, 50]. Data on attributable mortality remain limited. In a study from Russia involving 38 patients with *C. auris* candidemia, the crude mortality rate was 39.5%, while the attributable mortality rate was reported as 0% [51]. Due to the retrospective design of our study, attributable mortality could not be determined. Furthermore, several studies have noted that calculating attributable mortality is particularly challenging due to the high frequency of ICU admissions and comorbidities in this patient population.

Currently, neither the Clinical and Laboratory Standards Institute (CLSI) nor the European Committee on Antimicrobial Susceptibility Testing (EUCAST) has established official antifungal susceptibility breakpoints for *C. auris.* Therefore, the CDC recommendations are followed for a limited number of agents (amphotericin B, fluconazole, and echinocandins). The CDC recommends echinocandins as the first-line treatment and lipid formulation amphotericin B as an alternative; however, it does not strictly restrict the use of azoles [27]. In cases of PDR *C. auris*, treatment options become even more limited [52]. Moreover, the correlation between MIC values and clinical outcomes has not been clearly established, which hinders the development of a consensus on susceptibility breakpoints for *C. auris* [25, 53]. These issues highlight the need for further research on treatment strategies in critically ill patients with *C. auris* infections. Each case of candidemia represents valuable clinical data that may contribute to the development of an international consensus on the management of *C. auris.* In our study, resistance rates were as follows: 96% for fluconazole, 18.2% for amphotericin B, 8% for caspofungin, and 0% for micafungin. The rate of MDR was 13%, and the rate of PDR was 8.7%. Antifungal resistance rates reported in various studies are summarized in Table 4. Consistent with previous literature, the highest resistance rate was observed for fluconazole. However, the PDR rate identified in our study was considerably higher than those reported in the literature, underscoring the critical importance of strict infection control measures.

**Table 4.** Comparison of *C. auris* antifungal resistance, MDR, and PDR rates with previous studies

Resistance/country, reference	Fluconazole (%)	Amphotericin B (%)	Caspofungin (%)	Micafungin (%)	MDR (%)	PDR (%)
Our study	96	18.2	8	0	13	8.7
Chowdhary et al. (IN, 2014) [54]	100		40			
Lockhart et al. (Multinational; PK, IN, ZA, VE, 2017) [13]	93	35			41	4
Sayeed et al. (PK, 2019) [33]	100	7.9	0	0	4.76	0
Arensman et al. (USA, 2020) [55]	14	3.6			-	3.6
Shastri et al. (North India, 2020) [34]	97	93.7	3	0	-	-
Chen et al. (Review, 2020) [19]	91	12	12.1 (for India 23.6%)	0.8	-	-
Pandya et al. (Multinational, IN, TR, OM, USA, IT, PK, BH, 2021) [56]	78	58	16	0	-	-
Simon et al. (USA, 2023) [57]	98.3	86.4		0	-	-
Koleri et al. (QA, 2023) [47]	91	84.8	9	0	-	-
Munshi et al. (SA, 2024) [35]	97.8	17.4	4.3	4.3	-	-
Erdem et al. (Multinational, TR, IN, SA, EG, BH, BD, AF, 2025) [50]	90.4	62.7	9	0.8	58.4	0.8

Abbreviations: AF, Afghanistan; BD, Bangladesh; BH, Bahrain; EG, Egypt; IN, India; IT, Italy; MDR, Multidrug resistance; OM, Oman; PDR, pandrug resistance; PK, Pakistan; QA, Qatar; SA, Saudi Arabia; TR, Türkiye; USA, United States of America; VE, Venezuela; ZA, South Africa.

Our study has several limitations. First, its retrospective design and the lack of analysis regarding the genetic basis of antifungal resistance and virulence-related characteristics limit the scope of interpretation. Additionally, the modest sample size may have influenced the results and contributed to the wide CIs observed. However, a post hoc power analysis demonstrated acceptable statistical power (>70%) for most significant associations, supporting the robustness of our findings despite the limited sample size. This may explain why some variables did not reach statistical significance despite apparent trends. Nevertheless, the inclusion of a control group strengthens the study by enabling a clearer assessment of the true impact of the infection and facilitating the identification of potential causal relationships. Furthermore, it is important to emphasize the need for reliable antifungal susceptibility testing to guide clinical decision-making, as variations in testing methods and limited availability of reference techniques may affect the interpretation of resistance data.

*C. auris* is a high-mortality pathogen that places an additional burden on healthcare systems and remains at the forefront of the global antimicrobial resistance (AMR) agenda. It is frequently encountered in healthcare settings, particularly among patients with comorbidities. The organism can persist on medical devices and in the hospital environment for extended periods. With increasing reports of pan-resistance, therapeutic options are becoming progressively limited. There is an urgent need for a comprehensive intervention strategy to reduce the burden of *C. auris.* In addition, timely removal of CVCs, whenever feasible, should be emphasized as an important preventive measure against candidemia, as CVC presence was identified as an independent risk factor in this study. This programme should include robust surveillance systems to monitor clinical, epidemiological, and antifungal resistance patterns, and it must reinforce infection control practices within hospitals. In our study, broad-spectrum antibiotic use was associated with a 46-fold increased risk of *C. auris* candidemia; however, this estimate carries uncertainty due to the small sample size and possible multicollinearity among predictors. Our findings emphasize the necessity for early identification, strict antimicrobial stewardship, and device management strategies such as timely CVC removal in high-risk hospital units.

## Data Availability

All data generated or analysed during this study are included in this published article. The datasets used and/or analysed during the current study are available from the corresponding author on reasonable request.
